# Bioinformatic Tools for Inferring Functional Information from Plant Microarray Data: Tools for the First Steps

**DOI:** 10.1155/2008/147563

**Published:** 2008-05-29

**Authors:** Grier P. Page, Issa Coulibaly

**Affiliations:** Department of Biostatistics, University of Alabama at Birmingham, 1665 University Blvd Ste 327, Birmingham, AL 35294-0022, USA

## Abstract

Microarrays are a very powerful tool for quantifying the amount of RNA in samples; however, their ability to query essentially every gene in a genome, which can number in the tens of thousands, presents analytical and interpretative problems. As a result, a variety of software and web-based tools have been developed to help with these issues. This article highlights and reviews some of the tools for the first steps in the analysis of a microarray study. We have tried for a balance between free and commercial systems. We have organized the tools by topics including image processing tools (Section 2), power analysis tools (Section 3), image analysis tools (Section 4), database tools (Section 5), databases of functional information (Section 6), annotation tools (Section 7), statistical and data mining tools (Section 8), and dissemination tools (Section 9).

## 1. INTRODUCTION

The primary goal of a microarray study is to generate a list of differentially regulated genes and infer pathways that can provide insight into the biological question under investigation. Due to the very high dimensionality of a microarray experiment, running to thousand of genes, bioinformatics, and statistical tools are essential for the analysis of data. This review is written to provide plant investigators with a list of tools and web-based resources designed to help them move from an idea or hypothesis to the conduct of the study, image analysis, generation of expression data, statistical analysis, annotation, and then dissemination of the data.

The first step in the conduct of a microarray study is the selection of a microarray platform to use. For many species, there are commercially available arrays from commercial vendors and academic groups. Unfortunately, arrays are not available for all species, while arrays can be used in closely related species, it is usually better to develop arrays based upon the sequence of the species being studied. Section 2 provides a list of tools for generating useful probe sequences from genomic data. Once an array has been developed, it is critical to collect sufficient samples to run an experiment that will generate biologically generalizable results. Section 3 highlights tools for sample size and power analysis for microarray studies. Image analysis tools (Section 4) are used to quantitate the amount of fluorescence for a spot or set of spots. Microarray experiments generate copious amounts of data. The storage and distribution of the data are accomplished by the tools described in Section 5. Databases of gene annotations are provided in Section 6. Sections 7 and 8 describe statistical analysis and annotation tools. The two grouped together for the same tools often provide both functions. In fact, many of the database tools will also provide analytical and annotation functions as well. Finally, in Section 9 we describe web sites for disseminating microarray data and analyses.

## 2. PROBE DESIGN SOFTWARE

Plant scientists conduct their research on a wide variety of plant taxa. Arrays have been developed for a number of plant species including Arabidopsis, Maize, Populus, Rice, Barley, Grape, Citrus, Cotton, Medicago, Soybean, Sugar Cane, Tomato, and Wheat. While arrays can be used on closely related species, it is often better to design a new array for the species of interest. Several tools have been designed to help design probes for spotting or deposition on arrays, based upon genomic sequence data. The critical stage is to have high-quality sequence data. The more complete the genome is, the easier it will be to design probes that will not cross hybridize, be subject to SNPs, and query the gene accurately. Table 1 lists a number of tools for probe design; many of them are free, but a number specific to a single array manufacturer.

## 3. POWER ANALYSIS AND SAMPLE SIZE CALCULATIONS

One of the keys to a successful microarray study is to collect enough data (arrays) in order to derive biologically generalizable results. The key to this is the statistical power of a study. Power is the probability of being able to detect a significant difference between experiment groups when one really exists. There are several factors involved in power, but the main one under the control of an investigator is the sample size. A study with too few samples may not detect real differences, while too many samples will waste resources. Power analysis allows the selection of the optimal sample size. While sample sizes for microarrays can be planned with traditional statistical power calculation tools such as PS (http://biostat.mc.vanderbilt.edu/twiki/bin/view/Main/PowerSampleSize), the unique features of arrays such as the large number of tests and the large number of genes that are different between groups have lead to the development of several methods and tools for calculating power and sample size analysis.

### 3.1. The Power Atlas

The Power Atlas is a web-based resource to assist investigators in the planning and design of microarray and expression-based experiments. This software currently aims at estimating the power and sample size for a two group comparison based upon pilot data. The methods underlying the web site are reported in Gadbury et al. [1] and the software is described in further detail at Page et al. [2]. The tool may be used in two manners: one may either upload one's own pilot data or select a pilot dataset from over 1 000 public data sets. Output includes graphs of power for a variety of significance and false discovery rates; see http://www.poweratlas.org/ [2].

### 3.2. Significance analysis of microarrays (SAM)

SAM is a free flexible Excel Addin that includes a number of useful functions for the analysis of microarray data. Tools include statistical analysis for discrete, quantitative, and time series data, adjustments for multiple testing, gene set enrichment analysis, sample size assessment, estimates of False Discovery rate (FDR) and *q*-value, as well as per gene power analysis; see http://www-stat.stanford.edu/~tibs/SAM/ [3].

## 4. IMAGE ANALYSIS SOFTWARE

The purpose of image analysis software is to generate a quantified expression score from the scanned microarray images. Some of the tools are specific to particular array types, and thus are not appropriate for all array types. There are a number of tools that are available in this area, many of which are expensive. We present here tools that are still being actively supported and developed. Additional tools are listed in Table 2.

### 4.1. Affy

This is a package in Bioconductor for processing Affymetrix arrays. A wide variety of image processing, normalization, and quality control procedures are available. As a note, there are a variety of other image processing tools in Bioconductor including PDNN and DCHip that should be considered for use as well; see http://www.bioconductor.org/packages/2.1/bioc/html/affy.html [4].

### 4.2. Affyprobe miner

Affyprobe miner is used to redefine chip definition files (CDFs) for Affymetrix chips to take into account the more recent genomic sequence information on SNP, alternative splicing, changes in the gene model, exon structure, and other such genomic difference. Precomputed CDFs for several chips are available for download; see http://gauss.dbb.georgetown.edu/liblab/affyprobeminer/ [5].

### 4.3. Beadarray

This is a function in Bioconductor for reading preprocessed Illumina Bead summary data as well as reconstructing bead-level data using raw TIFF images. Methods for quality control and low-level analysis are also provided; see http://www.bioconductor.org/packages/2.1/bioc/html/beadarray.html [6].

### 4.4. Genechip operating software (GCOS)

Affymetrix GCOS automates the control of GeneChip Fluidics Stations and Scanners. In addition, GCOS acquires data, manages sample and experimental information, and performs gene expression data analysis. GCOS can quantitate images using MAS 5 and PLIER; see http://www.affymetrix.com/products/software/specific/gcos.affx.

### 4.5. Gene pix pro 6.0

This software has a number of useful features including imaging, spot finding, quality control, analysis tools, visualizations, and automation capabilities. GenePix can display and process up to four single wavelengths, thus four-channel imaging can be used. This tool can be integrated with a web-accessible database. GenePix is in some ways the default industrial standard microarray image analysis software because of its early development of couple of output file formats, ∗.gpr and ∗.gps that are used by many other applications; see http://www.moleculardevices.com/.

### 4.6. Nimblescan

This is a NimbleGen product designed for the extraction of feature intensity raw values, linkage of the raw intensity values with the corresponding probe parameters, and generation of analysis reports for expression, ChIP-chip and resequencing arrays, and methylation analysis for NimbleGen Arrays; see http://www. nimblegen.com/products/software/nimblescan.html.

### 4.7. TM4/spotfinder

Spotfinder is part of the larger freely available microarray analysis suite TM4. Spotfinder is designed for the rapid, reproducible, and computer-aided analysis of microarray images, and the quantification of gene expression. Spotfinder can read paired 16-bit or 8-bit TIFF image files generated by most microarray scanners. Automatic, semiautomatic and manual grid construction and adjustments can be made. Two segmentation methods are available. Reusable grid geometry files and automatic grid adjustment allow user to analyze large quantities of images in a consistent and efficient manner. Quality control views allow the user to assess systematic biases in the data; see http://www.tm4.org/spotfinder.html [7, 8].

## 5. DATABASE TOOLS

Microarray experiments generate a huge amount of data. The handling, storing, sharing, and distribution of the data can be quite complex. As a result a variety of database tools have been developed for assisting in this aspect of microarray studies. Some of the tools listed below are more than just stand-alone database tools and may include extensive analysis and visualization functionality as well. There are a number of database tools with highly different utility and platform requirements. Table 3 outlines the tools and websites.

## 6. DATABASES OF FUNCTIONAL INFORMATION

The amount of information about the functions of genes is beyond what any one person can know. Consequently, it is useful to pull in information on what others have discovered about genes in order to fully and correctly interpret an expression study. The following tools are databases of various types on information such as published papers, gene sequences, pathways, and ontologies that might be useful for an investigator who is interpreting an expression study.

### 6.1. Agbase

AgBase is a curated, open-source, web-accessible resource for functional analysis of agricultural plant and animal gene products. Agbase contains databases of Poplar and Pine gene ontology terms and annotations as well as several animals, microbes, and parasites; see http://www.agbase.msstate.edu) [9, 10].

### 6.2. Agricola

Agricola is the catalog and index to the collections of the National Agricultural Library. The database covers materials in all formats and periods, dating back to the 15th century. The records include all aspects of agriculture and related disciplines; see http://agricola.nal.usda.gov/.

### 6.3. Eukaryotic gene orthologues (EGO)

EGO is generated by the pair-wise comparison between the tentative consensus (TC) sequences from individual organisms. The reciprocal pairs of the best match are clustered into individual groups and multiple sequence alignments are displayed for each group. EGO is very useful for connecting homologous genes across species; see http://compbio.dfci.harvard.edu/tgi/ego/ [11].

### 6.4. Ensembl

Ensembl is a joint project between European Bioinformatics Institute and the Wellcome Trust Sanger Institute to develop a software system which produces and maintains automatic annotation on selected eukaryotic genomes. Initially developed for vertebrates, Ensembl has been adapted for use by several plant groups including legume, Gramene, and Arabidopsis; see http://www.ensembl.org/index.html [12].

### 6.5. Entrez gene

Entrez Gene is an NCBI's database for gene-specific information. Entrez Gene focuses on the genomes that have been completely sequenced, have an active research community to contribute gene-specific information, or that are scheduled for intense sequence analysis. Records are assigned unique, stable and tracked integers as identifiers. The content (nomenclature, map location, gene products and their attributes, markers, phenotypes, and links to citations, sequences, variation details, maps, expression, protein homologs, protein domains and external databases) is updated regularly. There is currently at least some gene information on 113 plant species; see http://www.ncbi.nlm.nih.gov/sites/entrez?db=gene.

### 6.6. Gene index

The goal of The Gene Index Project is to use the available EST and gene sequences, along with the reference genomes, to provide an inventory of likely genes and variants. Genes are linked to annotation regarding their functions. Currently GI databases have been constructed for 34 plant species; (http://compbio.dfci.harvard.edu/tgi/plant.html) [13, 14].

### 6.7. Gene ontology

The objective of GO is to provide controlled vocabularies for the description of the molecular function, biological process, and cellular component of gene products. These terms are to be used as attributes of gene products by various collaborating databases such as Gramene and TAIR; see http://www.geneontology.org/ [15].

### 6.8. Gramene

Gramene is a curated, open-source, data resource for genome analysis in the grasses. The information stored in the database is derived from public sources and includes genomes, EST sequencing, protein structure and function analysis, genetic and physical mapping, interpretation of biochemical pathways, Gene Ontologies, gene and QTL localization and descriptions of phenotypic characters and mutations. Extensive information is provided for *Oryza, Zea, Triticum, Hordeum, Avena, Setaria, Pennisetum, Secale, Sorghum, Zizania,* and *Brachypodium*; see http://www.gramene.org/.

### 6.9. Kyoto encyclopedia of genes and genomes (KEGG)

KEGG is a database of biological systems, consisting of genes and proteins (KEGG GENES), endogenous and exogenous substances (KEGG LIGAND), pathways (KEGG PATHWAY), and hierarchies and relationships of biological objects (KEGG BRITE). This database is very rich in data with information across hundreds of species including many plants; see http://www.genome.jp/kegg/ [16–18].

### 6.10. Plant associated microbe geneontology (PAMGO)

PAMGO is a database of the results of a multiinstitutional collaborative effort, aimed at developing new GO terms and relationships for gene products implicated in plant-pathogen interactions. GO terms are currently being developed for the following species: *Erwinia chrysanthemi*, *Pseudomonas syringae* pv tomato and *Agrobacterium tumefaciens*, the fungus *Magnaporthe grisea*, the oomycetes *Phytophthora sojae* and *Phytophthora ramorum*, and the nematode *Meloidogyne hapla*; *see* http://pamgo.vbi.vt.edu/.

### 6.11. SWISS-PROT

SWISS-PROT is a curated protein sequence database which provides high level of annotations such as the description of the function of a protein, its domains structure, post-translational modifications, variants, and so forth, along with good integration with other databases; see http://www.expasy.ch/sprot/.

### 6.12. TAIR

The Arabidopsis Information Resource (TAIR) maintains a database of genetic and molecular biology data for *Arabidopsis thaliana.* Data available from TAIR includes the complete genome sequence along with gene structure, gene product information, metabolism, gene expression, DNA and seed stocks, genome maps, genetic and physical markers, and publications; see http://www.arabidopsis.org/.

## 7. ANNOTATION TOOLS

The databases described in Section 6 can provide data in a variety of forms, which makes merging the annotations with the expression difficult. To deal with this heterogeneity a number of tools have been developed to increase the ease of annotating genes in expression studies.

### 7.1. CiteXplore

CiteXplore combines literature search with text mining tools for biology. Search results are cross referenced to European Bioinformatics Institute applications based on publication identifiers. Links to full text versions are provided where available; see http://www.ebi.ac.uk/citexplore/.

### 7.2. Database for annotation, visualization, and integrated discovery (DAVID)

DAVID provides a huge set of functional annotation tools for investigators to understand biological meaning behind a large list of genes. The key is the DAVID Knowledgebase which provides a comprehensive, high-quality collection of gene annotation resource, the flexibility to cross-reference gene identifiers, and heterogeneous annotations from almost all databases. The DAVID tools are able to identify enriched biological themes, particularly GO terms, cluster redundant annotation terms, visualize genes on Baccarat and KEGG pathway maps, display related many-genes-to-many-terms on 2D view, search for other functionally related genes not in the list, list interacting proteins, highlight protein functional domains and motifs, redirect to related literatures, and convert gene identifiers from one type to another; see http://david.abcc.ncifcrf.gov/ [19].

### 7.3. MatchMiner

MatchMiner translates between several gene identifier types for the same list of hundreds or thousands of genes. The gene identifier types supported by the tool includes GenBank accession numbers, IMAGE clone IDs, common gene names, HUGO names, gene symbols, UniGene clusters, FISH-mapped BAC clones, Affymetrix identifiers, and chromosome locations. MatchMiner can also find the intersection of two lists of genes specified by different identifiers; see http://discover.nci.nih.gov/matchminer/index.jsp [20].

### 7.4. Medminer

MedMiner searches and organizes the biomedical literature on genes, gene-gene relationships, and gene-drug relationships. It uses GeneCards, PubMed, and syntactic analysis, truncated-keyword filtering of relational and user-controlled sculpting of Boolean queries to generate key sentences from pertinent abstracts. Abstracts selected can be automatically entered into EndNote; see http://discover.nci.nih.gov/textmining/main.jsp [21].

## 8. DATA ANALYSIS SOFTWARE

There is an incredible breadth of tools in this area with many tools providing very slick interfaces and very useful functions; however, you really do not need any of these tools. Most statistical packages such as SAS, SPSS, JMP, and R can be used to analyze microarray data and will do most of the functions the following tools will do, for there are few statistical methods that are 100% unique to expression studies. Nonetheless many of the following tools are much easier to use and often have better visualization functions than the pure statistical programs. Typically the tools have been designed for ease of use, often too easy. Regardless of the tool you use, strive to understand the function and analyses provided and the assumption that are made when you choose to use them for analysis. For example, in cluster analysis you need to make a choice of link and weight functions and the clusters that result will be quite different based on methods which are chosen. There are similar issues to learn and understand for all statistical methods and most visualization methods. Additional tools are listed in Table 4.

### 8.1. Bioconductor

Bioconductor is a multicenter effort to develop tools in the R programming environment for analyzing genomic data, especially microarray data. There are a large number of different packages available to conduct many types of analyses; currently there are over 115 microarray applications. Tools are still in very active development, and are all freely available. Some of the most relevant tools are affy, maanova, genefilter, limma, mulltest, annotate, geneplotter, marray to name a few. A couple of the packages are described elsewhere in this document, but for more details of specific tools see the Bioconductor web site; see http://www.bioconductor.org/ [22].

### 8.2. Biometric research branch (BRB) arrays tools

BRB ArrayTools is a free integrated package for the visualization and statistical analysis of DNA microarray gene expression data. It functions as an Excel Addin. It was developed by professional statisticians experienced in the analysis of microarray data. It is probably the best tool available for discriminate analysis and has a variety of other statistical and cluster methods included; see http://linus.nci.nih.gov/BRB-ArrayTools.html.

### 8.3. Expression profiler

Expression Profiler is a set of tools for cluster analysis, pattern discovery, pattern visualization, study and search for gene ontology categories. The tool also generates sequence logos, extracts regulatory sequences, studies protein interactions, and links analysis results to external tools and databases; see http://ep.ebi.ac.uk/.

### 8.4. Genepattern

 GenePattern puts sophisticated computational methods into the hands of the biomedical research community. A simple application interface gives a broad audience access to a growing repository of analytic tools for genomic data, while an API supports computational biologists. GenePattern is a powerful analysis workflow tool developed to support multidisciplinary genomic research programs and designed to encourage rapid integration of new techniques; see http://www.broad.mit.edu/cancer/software/genepattern/index.html [23].

### 8.5. GeneXpress

GeneXPress is a visualization and analysis tool for gene expression data, integrating clustering, gene annotation, and sequence information. GeneXPress allows the user to load clustering results and automatically analyze them for significance of functional groups through correlation with functional annotations (e.g., Gene Ontology) and for enrichment of motif binding sites (e.g., TRANSFAC motifs); see http://genexpress.stanford.edu/.

### 8.6. GEPAS (gene expression pattern analysis suite)

GEPAS is an integrated web-based tool for the analysis of gene expression data. GEPAS includes tools for normalization, many clustering methods, supervised analysis, differential analysis, differential gene expression, predictors, array CGH and functional annotation; see http://gepas.bioinfo.cipf.es/ [24, 25].

### 8.7. High-dimensional biology statistics (HDBStat!)

HDBStat is a free java application that allows for the normalization, transformation, and statistical analysis of expression data. HDBStat also has a number of unique quality control procedures included. HDBStat has implemented reproducible research design to allow for analysis to be readily repeated; (http://www.ssg.uab.edu/hdbstat/) [26].

### 8.8. JMP genomics

JMP genomics leverages many statistical tools in JMP, a statistical analysis package, as a result it has over 100 di- fferent analytical procedures that can be run. It also includes extensive visualization tools. Scripts can be written for the development of standard analytical procedures; see http://www.jmp.com/software/genomics/.

### 8.9. Onto-tools

Onto-Tools are a series of freely available tools for the analysis of microarray data. Tools are available for array design (onto-design), gene class testing (onto-express), comparing the content of arrays (onto-compare), mapping gene information across databases (onto-translate), annotation (onto-miner), and pathway analysis (pathway-express); see http://www.vortex.cs.wayne.edu [27].

### 8.10. Partek genomic suite

Partek Genomics Suite can be used for gene expression analysis, exon expression analysis, chromosomal copy number analysis, and promoter tiling array analysis, and analysis of SNP arrays. Partek includes a large number of statistical, visualization, and annotation tools that can be tied together using workflow tools for rapid repetition of analysis and for reproducible research; see http://www.partek.com/software/.

### 8.11. R/maanova

Maanova stands for MicroArray ANalysis Of VAriance. It provides a complete work flow for microarray data analysis including data-quality checks and visualization, data transformation, ANOVA model fitting for both fixed and mixed effects models, statistical tests including permutation tests, confidence interval with bootstrapping, and cluster analysis. R/maanova is available in Bioconductor/R; refer to http://www.jax.org/staff/churchill/labsite/software/ Rmaanova/index.html [28].

### 8.12. SAM (significant analysis of microarrays)

SAM can be used on any type of array data: oligo or cDNA arrays, SNP arrays, protein arrays, and so forth. Both parametric and nonparametric tests are available for correlating expression data to clinical parameters including treatment, diagnosis categories, survival time, paired data, quantitative (e.g., tumor volume), and one-class. SAM can also implement imputation methods for missing data via nearest neighbor algorithm; see http://www-stat.stanford.edu/~tibs/SAM/.

### 8.13. TM4

The TM4 suite of tools consists of four major applications, Microarray Data Manager (MADAM), TIGR_Spotfinder, Microarray Data Analysis System (MIDAS), and Multiexperiment Viewer (MeV), as well as a MySQL database, all of which are freely available. Although these software tools were developed for spotted two-color arrays, many of the components can be easily adapted to work with single-color formats such as filter arrays and GeneChips; see http://www.tm4.org/index.html.

## 9. DISSEMINATION

Early in the use of microarray in research, it became common practice for many journals to require investigators to submit expression data for publication in a public database. This sharing of data has allowed the mining of these rich resources that many investigators have used to help their research. A number of the public databases exist that contain and accept plant data.

### 9.1. ArrayExpress

ArrayExpress is a public repository for microarray data, which is aimed at storing MIAME-compliant data in accordance with MGED recommendations. This database is a bit less biomedical in focus than GEO with a good representation of plant expression data; see http://www.ebi.ac.uk/arrayexpress [29, 30].

### 9.2. GEO

Gene Expression Omnibus is a gene expression/molecular abundance repository supporting MIAME compliant data submissions, and a curated, online resource for gene expression data browsing, query and retrieval. This is supported by the US National Library of Medicine, but contains a good amount of plant expression data; see http://www.ncbi.nlm.nih.gov/projects/geo/ [31, 32].

### 9.3. NASC (nottingham arabidopsis stock center) arrays

NASC runs a database of its own arrays as well as other data that has been deposited in the database. The database primarily contains Arabidopsis array data; see http://affymetrix.arabidopsis.info/ [33].

### 9.4. Plant expression database (PlexDB)

PLEXdb is a unified public resource for gene expression for plants and plant pathogens. PLEXdb serves as a portal to integrate gene expression profile data sets with structural genomics and phenotypic data. Data from seven species is contained in the database; see http://www.plexdb.org/index.php [34].

## 10. CONCLUSIONS

We hope this listing of tools, which only dip the surface of the possible tools, will assist you in conducting, analyzing, and interpreting expression studies. We suggest exploring several tools in an area and understanding the principles of the methods implemented before settling on one or a few to use regularly. By exploring several tools you will understand the potential of the various tools, how easy (or difficult) they are to use, and determine what you really want and need for your microarray analysis.

## Figures and Tables

**Table 1:** Probe design software packages.

Tool and website	Cost and functions of the tool
Array Designer http://www.premierbiosoft.com/dnamicroarray/index.html	Design primers and probes for oligo and cDNA expression microarrays. It can also design probes for SNP detection, single exon, whole gene, tiling, and resequencing arrays. The software is not free.
ArrayScribe http://www.nimblegen.com/products/software/arrayscribe.html	Free, but limited to designing NimbleGen Arrays. The tool can design probes, specify mismatches at specific sequence positions, automatically generate mismatches, generate multiple probes for a gene, and design the placement of spots on an array.
eArray http://earray.chem.agilent.com/earray/login.do	Free, but limited to designing Agilent arrays. Can design probes for expression, CGH, and ChiP for any species with genomic sequence.
Primer3Plus http://www.bioinformatics.nl/cgi-in/primer3plus/primer3plus.cgi	Free software that can design probes for expression detection on arrays, amplification/cloning, and sequencing/resequencing.
Sarani Oligo Design http://www.strandls.com/oligodesign.html	Probe design for expression analysis. The software is not free.
Visual OMP http://www.dnasoftware.com/Products/VisualOMP	Design software for RNA, DNA, single or multiple probe design, microarrays, TaqMan assays, genotyping, single and multiplex PCR, secondary structure simulation, sequencing, genotyping.

**Table 2:** Other useful image analysis software packages.

Tool name	Web site
Able Image Analyser	http://able.mulabs.com/
ArrayVision	http://www.imagingresearch.com/products/ARV.asp
IcononClust	http://www.clondiag.com/frame.php?page=/products/sw/iconoclust/index.php
ImaGene	http://www.biodiscovery.com/index/imagene
Koadarray	http://www.koada.com/koadarray/
Microvigene	http://www.vigenetech.com/MicroVigene.htm
ScanAlyze	http://rana.lbl.gov/EisenSoftware.htm
Spot	http://www.hca-vision.com/productspot.html

**Table 3:** Database tools.

Tool name	Web site
Acuity	http://www.moleculardevices.com/pages/software/gnacuity.html
Array Results Manager ARM	http://www.biodiscovery.com/index/arm
Arraytrack	http://www.fda.gov/nctr/science/centers/toxicoinformatics/ArrayTrack/ [35, 36]
BASE 2	http://base.thep.lu.se/
caArray	http://caarray.nci.nih.gov/
Expressionist	http://www.genedata.com/products/expressionist/index_eng.html
Gene Array Analyzer Software GAAS	http://www.medinfopoli.polimi.it/GAAS/
GeneDirector	http://www.biodiscovery.com/index/genedirector
GeneSpring Workgroup	http://www.chem.agilent.com/scripts/pds.asp?lpage=34668
GeneTraffic	http://www.iobion.com/products/products_GENETRAFFIC.html
Genowiz	http://www.ocimumbio.com/
Longhorn Array Database LAD [37]	http://www.longhornarraydatabase.org/
MaxdLoad2	http://www.bioinf.man.ac.uk/microarray/maxd/index.html
PARTISAN arrayLIMS	http://www.clondiag.com/
Rosetta Resolver System	http://www.rosettabio.com/products/resolver/default.htm
Stanford Microarray Database SMD	http://smd-www.stanford.edu//download/ [38]

**Table 4:** Other useful statistical analysis and data-mining tools.

Tool name	Web site
Amiada (analyzing microarray data)	http://dambe.bio.uottawa.ca/amiada.asp [39]
ArrayAssist Enterprise	http://www.stratagene.com/
caGEDA	http://bioinformatics.upmc.edu/GE2/GEDA.html
Cluster	http://rana.lbl.gov/EisenSoftware.htm
dChip	http://www.dchip.org/
GeneMaths XT	http://www.applied-maths.com/genemaths/genemaths.htm
INCLUSive	http://homes.esat.kuleuven.be/~dna/Biol/Software.html
J-Express Pro	http://www.molmine.com/software.htm
MAExplorer	http://maexplorer.sourceforge.net/
NIA Array analysis	http://lgsun.grc.nia.nih.gov/ANOVA/
Onto-Tools	http://vortex.cs.wayne.edu/projects.htm
Probe Profiler	http://www.corimbia.com/Pages/ProductOverview.htm
TableView	http://ccgb.umn.edu/software/java/apps/TableView/
Venn Mapper	http://www.gatcplatform.nl//vennmapper/index.php
